# Studies on the Mechanism of Alloimperatorin on the Proliferation and Apoptosis of HeLa Cells

**DOI:** 10.1155/2021/6617312

**Published:** 2021-04-08

**Authors:** Yingying Bai, Lijuan Yang, Chaihong Zhang, Yongxiu Yang

**Affiliations:** ^1^The First Clinical Medical College of Lanzhou University, Lanzhou, Gansu, China; ^2^Department of Obstetrics and Gynecology, First Hospital of Lanzhou University, Lanzhou, Gansu, China

## Abstract

Alloimperatorin is a compound extracted from the traditional Chinese medicine (Angelica dahurica), which has exhibited anticancer activity. However, its precise molecular mechanism of anticancer remains unclear. Alloimperatorin-induced apoptosis of cervical cancer cells and its molecular mechanism were investigated in the present study. Cholecystokinin octapeptide (CCK-8) was employed to evaluate the cytotoxicity of alloimperatorin on HeLa, SiHa, and MS-751 cells. Flow cytometry was used to assess apoptosis induced by alloimperatorin. The mechanism of apoptosis was verified by mitochondrial membrane potential, Western blotting, and fluorescent PCR. The results of the study showed that alloimperatorin reduced the activity of HeLa cells. The calculated IC_50_ at 48 hours was 116.9 *μ*M. Compared with the control group, alloimperatorin increased the apoptotic rate of HeLa cells and reduced the mitochondrial membrane potential of HeLa cells. The Western blot results showed that alloimperatorin promotes the expression of caspase3, 8, 9 and that Bax apoptotic proteins reduce PARP expression, procaspase3, 8, 9, and BCL-2 proteins and reduces the cyt-c in the mitochondria expression. The results demonstrated that alloimperatorin can induce HeLa cell apoptosis through mitochondria and extrinsic apoptotic pathways.

## 1. Introduction

Cervical cancer is a common malignant tumor in women. It has a high fatality rate and seriously endangers women's health [1]. The current first-line chemotherapeutic drugs for cervical cancer have made significant progress in its treatment. However, their strong side effects limit their use. Thus, there is an urgent need to use new compounds with lower side effects.

Inhibiting the proliferation of tumor cells and promoting their apoptosis is an important way to treat tumors, many studies have shown that apoptosis plays an important role in tumor therapy [2, 3]. Various anticancer drugs that promote apoptosis have been proven effective [4]. Thus, therapies that induce or promote apoptosis in cancer cells are important and are the subject of intense research.

In the exploration of anticancer drugs, natural drugs play an important role. Currently, commonly used anticancer drugs such as paclitaxel and vincristine are extracted from natural plants [5, 6]. The traditional Chinese medicine *Angelica dahurica* has antioxidant, anti-inflammatory, and antiviral activities [7–9]. It also shows anticancer activity in a variety of tumors [10–12]. There are a variety of extracted compounds from Angelica dahurica. Alloimperatorin is one of them [13, 14]. Some studies have shown that Alloimperatorin has anticancer activity in leukemia HL-60 cells [15]. However, the anticancer mechanism in cervical cancer is still elusive. In the present study, we investigated the antiproliferative effect of Angelica dahurica extract alloimperatorin on cervical cancer cells. Its mechanism is described.

## 2. Materials and Methods

### 2.1. Chemicals and Reagents

Alloimperatorin (98% pure) was purchased from Shaanxi Baoji Chenguang Co., Ltd., China, and dissolved in DMSO. The final concentration was 10 mg/ml and stored in a refrigerator at −20°C. The mother liquor was diluted with a basic medium to the required concentration for use. RPMI-1640, CCK-8 kit, caspase3, 8, 9, procaspase3, 8, 9, PARP, Bax, bcl-2 kits were all purchased from Abcam, Cambridge, MA.

### 2.2. Cell Culture

HeLa and SiHa cell lines were obtained from the Central Laboratory of the First Hospital of Lanzhou University. The MS-751 cell line was purchased from Shanghai Zhongqiao Xinzhou Biotechnology Co., Ltd., China. HeLa cells were cultured in DMEM medium with 10% fetal bovine serum, SiHa cells and MS-751 cells were cultured in MEM with 10% fetal bovine serum and incubated at 37°C (5% CO_2_).

### 2.3. Cell Viability Assay

The cell density consisted of (1 × 10^5^ cells/well planted and cultured in 96-well plates. The cells were then cultured for 24 hours to allow for growth and adherence to the wall. Alloimperatorin was subsequently added at a concentration of 5, 25, 50, 100, 150, and 200 *μ*M. The control group was grown in 0.1% DMSO. After 48 hours of treatment, 10 *μ*l of CCK-8 solution was added and incubated at 37°C and 5% CO_2_ for 1 hour. The OD values were detected at 450 nm with a microplate reader. The half-maximum inhibitory concentration (IC50) was calculated using Prism-GraphPad8.0 software. Cell viability (%) = [1–(ODcontrol−ODtreated)/(ODcontrol–ODblank)].

### 2.4. High Content Analysis (Perkin Elmer) System for Real-Time Monitoring of Cell Proliferation

A seeding density of 2,000 cells per well was grown in 96-well culture plates. The cells were divided into control (0.1% DMSO) and alloimperatorin (25, 50, 100, 150, and 200 *μ*M) treated groups. A PerkinElmer Operetta CLS high-content imaging analysis system was used for live cell dynamic monitoring. The cells were cultured at 37°C (5% CO_2_). A select digital phase contrast channel, a 10× objective lens, and full-hole shooting were used to monitor cell proliferation. The shooting was performed once every 1 h, for 66 h. Harmony software was used to measure dynamic cell proliferation for 66 h.

### 2.5. Staining Assessment

A seeding density of 1,000 cells per well was treated with either the control growth medium (0.1% DMSO) or alloimperatorin (50, 100, and 150 *μ*M) for 48 hours. The cells were subsequently washed with phosphate buffer. The JC-10 probe working solution was then added and incubated for 30 minutes. A photo was taken by the high content analysis system, and the number of green cells counted.

### 2.6. Apoptosis Assessment

After treating the cells with either alloimperatorin (50, 100, and 150 *μ*M) or 0.1% DMSO (control group) for 48 hours, the cells were collected and washed with phosphate-buffered saline (PBS) three times. The washed HeLa cells were then resuspended in 200 *µ*l of staining buffer and stained with 10 *µ*l annexin V-FITC (20 *µ*g/ml) and 5 *µ*l PI (50 *µ*g/ml) before SGC was performed. The HeLa cells were quantified using flow cytometry, and the CellQuest Pro 4.0 acquisition software (FACS Calibur; BD Biosciences, San Jose, CA, USA) is used for analysis.

### 2.7. Cell Cycle Assessment

HeLa cells were seeded in 6-well plates at a density of 1 × 10^6^/well and treated with either 0.1% DMSO or alloimperatorin (50, 100, and 150 *µ*M), respectively, for 48 hours. The cells were collected and fixed with 70% ethanol overnight. They were combined with RNase A at 37°C under dark conditions. The incubation was performed with 1 ml PI solution (20 *μ*g/ml 1% Triton X-100 in PBS) for 30 minutes. CellQuest software (BDIS) was used to evaluate the cell cycle by flow cytometry (BD Bioscience, MA, USA).

### 2.8. Mitochondrial Membrane Potential Assessment

HeLa cells were treated with alloimperatorin (50, 100, and 150 *µ*M) for 48 hours. The cells were then collected, washed with phosphate-buffered saline (PBS) three times, and 100 *µ*l of a JC-10 working solution was added and incubated at 37°C (5% CO_2_) for 15–60 minutes. A detection in fluorescence change was performed using a flow cytometer, and the fluorescence values of *Ex*/*Em* = 500/525 nm (FITC channel) and 540/595 nm (TRITC channel) recorded. The ratio of red fluorescence signal to the blue fluorescence signal was calculated and used to judge the health of the cells.

### 2.9. Wound Scratch Assay

A seeding density of 2 × 10^5^ cells was planted in a 24-well plate, using 500 *µ*l per well, and cultured 24 hours. When the cell growth and confluence were above 90%, a vertical scratch was made with the tip of a 200 *µ*l pipette tip and rinsed three times with PBS. The cell debris was subsequently removed, and the serum-free medium changed. Alloimperatorin (150 *µ*M) and control medium (0.1% DMSO) were added to the control and treatment group, respectively. They were then placed in a 37°C incubator for 24 hours. A high content analysis system (Perkin Elmer) was used to observe and take pictures of scratch wounds at 0, 6, and 24 hours. The experiment was repeated three times.

### 2.10. Western Blot Analysis

After treating HeLa cells (1 × 10^6^) with alloimperatorin (50, 100, and 150 *µ*M) for 48 hours, cell lysates were prepared and centrifuged at 12,000 × g at 4°C for 15 minutes. The total protein content was extracted from the supernatant, and the protein concentration was quantified by the bicinchoninic acid (BCA) assay method. The protein was separated at equal concentrations (30 *μ*g) with 10% SDS-polyacrylamide gel and then transferred to a PVDF membrane. After blocking with TBST containing 5% skim milk for 1 h, the membrane was incubated with rabbit monoclonal anti-GAPDH, caspase3, 8, 9 procaspase-3, procaspase-8, procaspase-9, Bcl-2, Bax, MMP-2, MMP-9, and PARP at 4°C overnight. The membrane was then washed with PBS and incubated with a secondary antibody conjugated with horseradish peroxidase (HRP) at 25°C for 1 hour. The proteins were visually detected with an enhanced chemiluminescence (ECL) kit.

### 2.11. Reverse Transcription-Polymerase Chain Reaction

Total RNA was extracted using TRIzol reagent. After quantifying RNA, a reverse transcription kit was used to synthesize cDNA to generate a 20 *µ*l reaction system. The primer sequence used was as follows. Bcl-2 forward: 5′-TGGACAACCATGACCTTGGAC-3′, reverse: 5′-GTGCTCAGCTTGGTATATGAGAA-3′; caspase8 forward: 5-′ATGAGCTGGGCTGAAGCAAAC-3′, reverse: 5′-AAGACCTCAATTCTGATCTGCTCAC-3′; caspase9 forward: AAGCCAACCCTAGAAAACCTTACC-3′, reverse: 5′-GACATCACCAAATCCTCCAGAAC-3′; BAX forward: 5′-CGACTGATGTCCCTGTCTCCA-3′, reverse: 5′-AGCACTCCCGCCACAAA-3′; caspase3 forward: 5′-GACTCTGGAATATAAATGGACAACA-3′, reverse: 5′-AGGTTTGCTGATCGACATCTG-3′, ACTB forward: 5′-TGGCACCCAGCACAATGAA-3, reverse: 5′-CTAAGTCATAGTCCGCCTAGAAGCA-3′; ACTB was used as an internal reference gene.

### 2.12. Statistical Analysis

All experiments were repeated three times. The statistical analysis was performed using the Prism-GraphPad8.0 software. All data were analyzed and represented as the mean ± SD of three experiments. A one-way ANOVA with Dunnet's posthoc test was used to compare the significant difference of alloimperatorin against the control group, with test *p* < 0.05 considered statistically significant.

## 3. Results

### 3.1. Alloimperatorin Inhibits the Proliferation of Cervical Cancer Cell Lines

**Figure 1:**
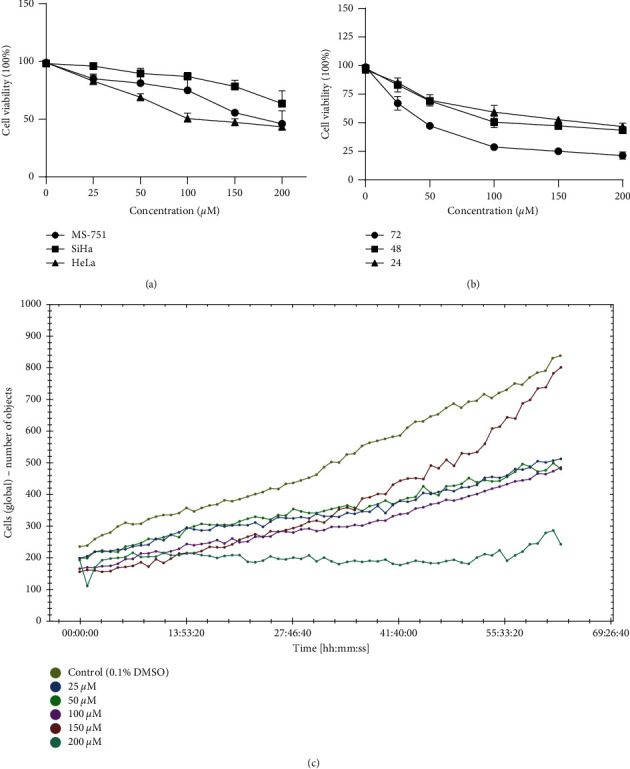
Inhibition of cell viability on cervical cancer cells. (a) The toxicity of alloimperatorin on HeLa, SiHa, and MS-751 cells was detected using the CCK-8 solution. (b) The inhibitory effect of alloimperatorin on HeLa cells at 24, 48, and 72 hours. (c) Alloimperatorin's antiproliferative effect on HeLa cells was continuously monitored using the high content analysis system (Perkin Elmer).

The toxicity of alloimperatorin to cervical cancer HeLa, SiHa, and MS-751 cells was tested with CCK-8.  Figure 1(a) shows the inhibitory effect of alloimperatorin on HeLa, MS-751, and SiHa cells. IC_50_ was 116.9, 148.0, and 324.5 *μ*M, respectively.  Figure 1(b) shows the inhibitory effect of alloimperatorin on HeLa cells at 24, 48, and 72 hours.  Figure 1(c) shows alloimperatorin's inhibitory effect on the proliferation of HeLa cells under continuous dynamic monitoring for 66 hours under the high content analysis system (Perkin Elmer). The results showed that alloimperatorin is very toxic on HeLa cells. Accordingly, we selected the HeLa cells for follow-up studies.

### 3.2. Alloimperatorin Induces HeLa Cell Apoptosis

**Figure 2:**
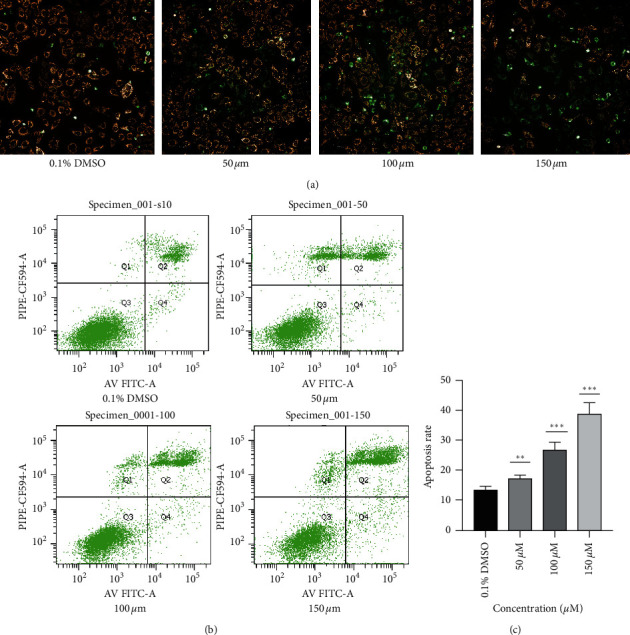
Alloimperatorin induced apoptosis in HeLa cells. (a) Alloimperatorin treated cells for 48 hours, stained with a JC-10 fluorescent probe. The number of green fluorescent cells was determined using a high content analysis system (Perkin Elmer). (b, c) HeLa cells were treated with alloimperatorin for 48 hours, double-stained with annexin V-FITC/PI, and apoptosis detected by flow cytometry. Data expressed as the mean ± SD of independent experiments, *n* = 3. Statistical analysis was performed using a one-way ANOVA test. ^*∗∗*^*p* < 0.01 compared to control.

The flow cytometry results of annexin V-FITC/PI double fluorescence staining (Figures 2(a)–2(c)) of HeLa cells treated with 0.1% DMSO (control group) and alloimperatorin (50 *μ*M, 100 *μ*M, and 150 *μ*M) for 48 hours showed that alloimperatorin significantly induced apoptosis by 17.8%, 24.5%, 35%, and 42%, respectively, compared to control.

### 3.3. Alloimperatorin Induced a Decrease in the Mitochondrial Membrane Potential of HeLa Cells

**Figure 3:**
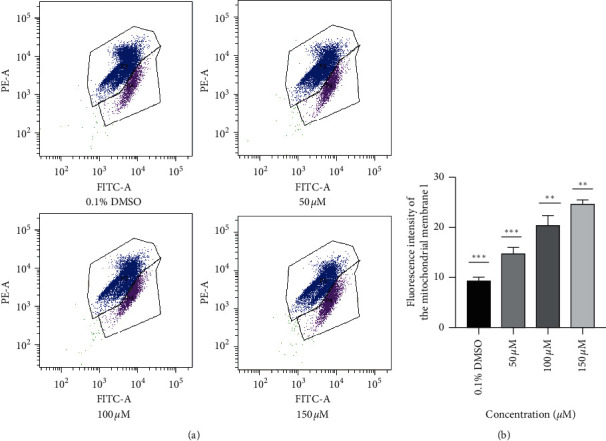
Alloimperatorin reduces cell mitochondrial membrane potential. (a) HeLa cells were incubated with alloimperatorin for 48 hours. Flow cytometry was used to detect mitochondrial membrane potential. (b) The data are expressed as the mean ± SD of independent experiments, *n* = 3, and statistical analysis was performed using a one-way ANOVA test. ^*∗∗∗*^*p* < 0.0001 compared to control.

The reduction of the mitochondrial membrane potential leads to the release of cytochrome C, which activates the proapoptotic protein. A flow cytometer was used to detect the fluorescence intensity of the mitochondrial membrane potential with a JC-10 probe. The experimental results demonstrated that the red fluorescence gradually increased with alloimperatorin concentrations. The results showed that alloimperatorin can decrease the mitochondrial membrane potential of HeLa cells in a concentration-dependent manner (Figures 3(a), and 3(b)).

### 3.4. Alloimperatorin Blocks HeLa Cell Cycle Arrest in the G1/S Phase

**Figure 4:**
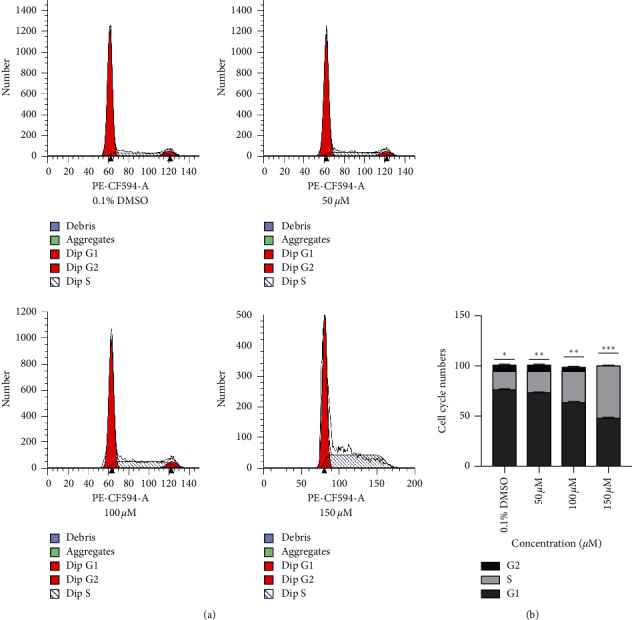
Cell cycle analysis of HeLa cells treated with alloimperatorin for 48 hours. (a, b). The value represents the percentage of the cell cycle. Each experiment was repeated three times.

HeLa cells were treated with different concentrations of alloimperatorin for 48 hours, and flow cytometry was used to test the distribution of the impact of alloimperatorin on the HeLa cell cycle by flow cytometry. The results (Figure 4(a)) showed that most of the cells were arrested in the G1 and S phases as the concentration increased. The proportion of the cells in the S phase increased, while the cells in the G2 phase gradually decreased. The data is expressed as the mean ± SD of independent experiments, *n* = 3. Statistical analysis was performed using a one-way ANOVA test. ^*∗∗∗∗*^*p* < 0.0001 compared to control at each timepoint (Figure 4(b)).

### 3.5. Alloimperatorin Inhibits Cell Wound Healing

**Figure 5:**
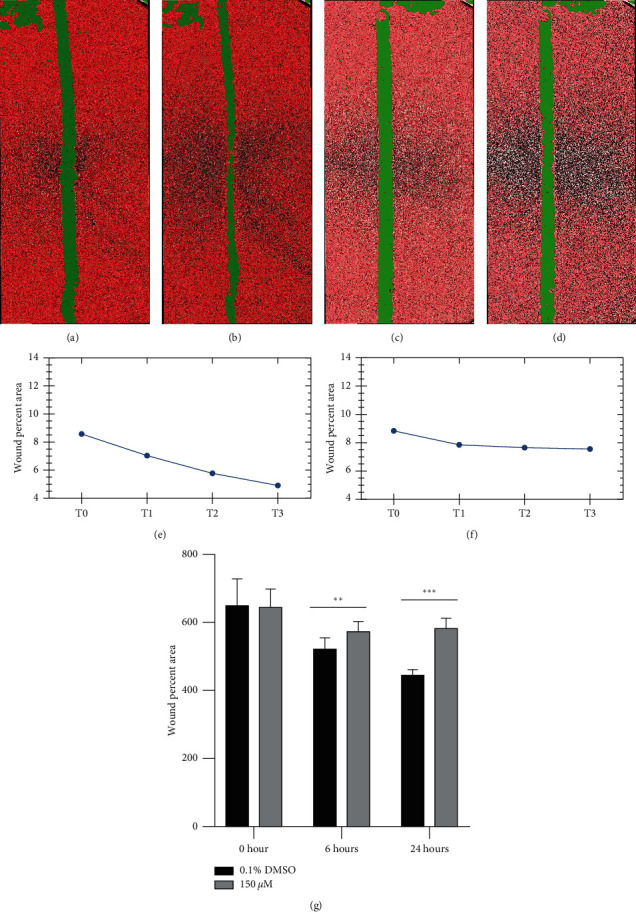
Alloimperatorin inhibited the migration of HeLa cells. (a–d) Alloimperatorin processed HeLa cells. The high content analysis system (Perkin Elmer) was used to detect cell scratch width and to take pictures. (e, f) Alloimperatorin treated cells were observed at 0, 6, 12, and 24-time points for the healing area. (g) The data are expressed as the mean ± SD of independent experiments, *n* = 3. The statistical analysis was performed using one-way ANOVA. ^*∗∗∗*^*p* < 0.0001 compared to control at each time point.

Figure 5 (a, b, c, d) and quantitative wound closure (E, F, G) are used for the healing test. HeLa cells were treated with either 150*μ*M alloimperatorin or 0.1% DMSO (control group). The high content analysis system (Perkin Elmer) was used to check the scratch width on cells at 0, 6, and 24 h. Alloimperatorin inhibited the migration of HeLa cells after 24 hours. The result shown in Figure 5(a) is the wound area of the control group at 0 hours, and the result shown in Figure 5(b) is the wound area at 24 hours.  Figure 5(c) is 0 hours in the drug group, and Figure 5(d) is the wound area in the drug group at 24 hours. Figures 5(e) and 5(f) are the wound area of the drug group and the control group at various timepoints, respectively. The results showed that the drug inhibited the healing of scratch wounds at each time point compared to the control group.

### 3.6. Alloimperatorin Promotes the Expression of Apoptotic Proteins and mRNA

**Figure 6:**
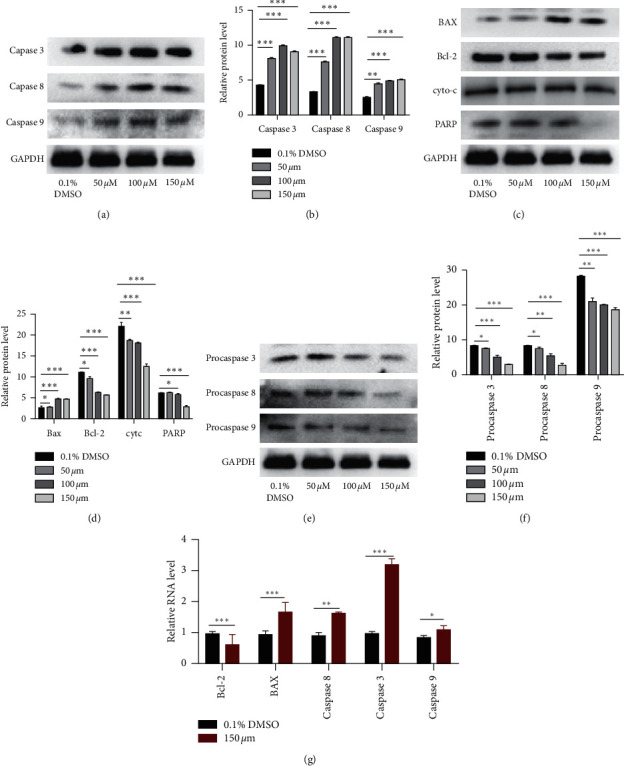
Alloimperatorin induces the expression of the apoptotic protein in HeLa cells. (a–e) Western blot was used to analyze alloimperatorin processed HeLa cells to detect the expression of Bcl-2, BAX, caspase3, 8, 9, procaspase3, 8, 9, PARP, and cy-tc. (f) Data are mean ± SD deviation from three independent experiments and are presented as fold change compared with control (^*∗∗*^*p* < 0.001 and ^*∗∗∗*^*p* < 0.0001 compared to control). (g) Fluorescence quantitative PCR was used to detect alloimperatorin-treated HeLa cells. The expression of caspase3, 8, 9, Bax, and bcl-2 mRNA in cells is shown.

At the protein level, various key effectors of apoptosis were studied to determine the mechanism of alloimperatorin-induced apoptosis. Caspase3 is a key factor in the process of apoptosis. The shearing of PARP marks the beginning of apoptosis and also the activation of caspase3. Our results show that HeLa cells treated with alloimperatorin can significantly reduce procaspase3, PARP, procaspases8 and 9, and Bcl-2 expression. On the contrary, the expression of caspase3, 8, 9 and Bax was significantly higher than that of the control group. Conversely, the cy-tc in the mitochondria was significantly lower than that of the control group (Figures 6(a) and 6(b)). The mRNA expression of caspase3, 8, 9 and BAX in alloimperatorin-treated HeLa cells was higher than that of the control group, while the mRNA expression of Bcl-2 was lower（Figure 6(f).

### 3.7. Alloimperatorin Inhibited Migration Protein Expression

**Figure 7:**
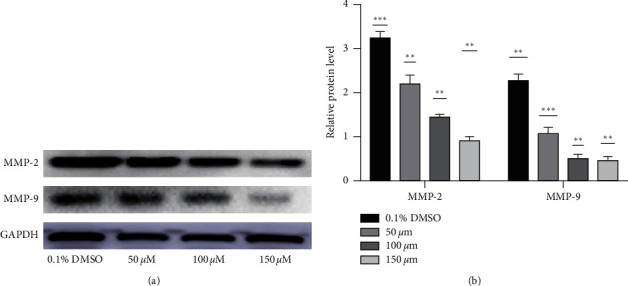
Alloimperatorin inhibited migration protein expression. HeLa cells were treated with different concentrations of alloimperatorin for 48 hours, and Western blot was used to test the expression of alloimperatorin on the HeLa cells. The results (a) showed that alloimperatorin inhibited the expression of migration protein, which has a clear trend compared with the control group. (b) Data are mean ± SD deviation from three independent experiments and are presented as fold change compared with control (^*∗∗*^*p* < 0.0001 compared to control).

Matrix metalloproteinase 2 (MMP-2) and matrix metalloproteinase 9 (MMP-9) play an important role in tumor invasion and migration .HeLa cells were treated with different concentrations of alloimperatorin for 48 hours, and western blot was used to test the expression of alloimperatorin on the HeLa cells .The results (Figures 7(a) and 7(b)) showed that alloimperatorin inhibited the expression of migration protein, which has a clear trend compared with the control group.

## 4. Discussion

Statistics from 2019 show that cervical cancer is still the second leading cause of death in women, with the disease occurring more frequently in younger women [16]. The current treatment of cervical cancer, including radiotherapy, chemotherapy, and targeted molecular therapy, has significantly improved the survival rate of patients. However, there are also significant side effects [17, 18]. In recent years, traditional Chinese medicine has made significant progress in cancer treatment [19, 20]. In our research, the CCK-8 experiment confirmed that alloimperatorin could effectively inhibit the proliferation of HeLa cells, and the inhibitory effect is more evident with the increase in concentration. The high content analysis system (Perkin Elmer) results after 66 hours of dynamic monitoring of cell proliferation showed that the alloimperatorin's inhibitory effect on HeLa cells is concentration-dependent. It is significant at 48 hours.

Apoptosis is programmed cell death involved in regulating the body's physiologic balance, shaping the development, and eliminating unnecessary cells in the body [21]. The reduction of apoptosis is associated with cancer development and can promote tumor progression [22–24]. In our study, the annexin V-FITC double-labeling method proved that compared to the control group, different concentrations of alloimperatorin could induce HeLa cells' apoptosis. The apoptosis rate was concentration-dependent. Mitochondria also play an important role in the apoptosis signaling pathway. When external factors stimulate cells, the mitochondrial membrane's permeability increases, and apoptotic factors are released, thereby triggering apoptosis [25]. Studies have shown that cells induced by different factors undergo apoptosis. When dying, it will cause mitochondrial dysfunction and a decrease in membrane potential [26]. In our study, it was confirmed by a JC-10 fluorescent probe that with the increase in alloimperatorin concentration, the red fluorescence of mitochondrial membrane potential gradually increased. The gradual decrease in membrane potential indicates that alloimperatorin is an apoptosis inducer and can promote the apoptosis of HeLa cells.

Dysregulation of the cell cycle is associated with tumor growth. Many anticancer drugs exert antitumor activity by blocking tumor cells at different stages, such as G1, S, or G2/M [27, 28]. In our current study, we used a drain cytometer to verify whether alloimperatorin can change the HeLa cells' cycle. The results showed that the percentage of the number of cells in the G1 phase and the number of cells in the G2 phase decreased gradually, and the number of cells in the S phase was gradually reduced. The percentage gradually increased, and most cells stagnated in the S phase. The results showed that alloimperatorin could block the HeLa cell cycle in the S phase, thereby inhibiting the growth of HeLa cells.

The apoptosis pathways of cells include endogenous (mitochondria), exogenous (death receptors), and endoplasmic reticulum apoptosis pathways, all of which depend on caspase activation. The Bcl-2 protein family controls the mitochondrial pathway. When external adverse factors stimulate cells, proapoptotic factors promote mitochondrial permeability, leading to the release of cytochrome c. This causes apoptotic bodies (cy-tc, caspase9, and Apaf-1) to form and activate the effector caspase (3, 6, 7) to perform apoptosis [29]. In this study, the results showed that in response to alloimperatorin, the expression of proapoptotic proteins Bax and caspase9 increased. Similarly, the expression of antiapoptotic protein bcl-2 decreased, the expressions of pro-caspase9 and PARP decreased, and procaspase9 and PARP expression decreased in HeLa cells. The cytochrome c expression decrease was significantly different from that of the control group. The results showed that alloimperatorin can induce HeLa cell apoptosis through the mitochondrial pathway. The external pathway (death receptor) can be stimulated by external factors to activate the death ligand (TRAIL/Apo2L) to activate further cell surface death receptors (DRs: TNF-R1, CD95, DR3, TRAIL-r1, TRAIL-r2, and DR6). This, in turn, stimulates the activation of caspase8 and initiates the proapoptotic cascade of caspase [30]. In this study, we found that alloimperatorin can activate the expression of caspase3, 8 protein and cause a decrease in the expression of procaspase3 and 8, showing a drug-dependent relationship, which was significantly different from the control group (Figure 6).

## 5. Conclusion

In this study, we found that alloimperatorin has a significant concentration-dependent toxic effect on HeLa cells. Alloimperatorin can also induce HeLa cells to undergo apoptosis, and the number of apoptosis gradually increases, showing apoptotic characteristics. Alloimperatorin can upregulate apoptotic proteins such as BAX, caspase3, 8, 9 and downregulate the expression of bcl-2, PARP, and procaspase3, 8, 9. These studies proved that alloimperatorin can induce HeLa cells' apoptosis through mitochondria and external pathways.

## Data Availability

All data in this study come from experimental operations. The data used to support the findings of this study are included within the article.
